# Carbonic Anhydrase Inhibitors Targeting Metabolism and Tumor Microenvironment

**DOI:** 10.3390/metabo10100412

**Published:** 2020-10-14

**Authors:** Andrea Angeli, Fabrizio Carta, Alessio Nocentini, Jean-Yves Winum, Raivis Zalubovskis, Atilla Akdemir, Valentina Onnis, Wagdy M. Eldehna, Clemente Capasso, Giuseppina De Simone, Simona Maria Monti, Simone Carradori, William A. Donald, Shoukat Dedhar, Claudiu T. Supuran

**Affiliations:** 1 Neurofarba Department, Pharmaceutical and Nutraceutical Section, University of Florence, Via Ugo Schiff 6, Sesto Fiorentino, 50019 Florence, Italy; andrea.angeli@unifi.it (A.A.); fabrizio.carta@unifi.it (F.C.); alessio.nocentini@unifi.it (A.N.); 2 IBMM, Univ. Montpellier, CNRS, ENSCM, 34296 Montpellier, France; jean-yves.winum@umontpellier.fr; 3 Latvian Institute of Organic Synthesis, Aizkraukles 21, 1006 Riga, Latvia, Institute of Technology of Organic Chemistry, Faculty of Materials Science and Applied Chemistry, Riga Technical University, 3/7 Paula Valdena Str., 1048 Riga, Latvia; raivis@osi.lv; 4 Computer-aided Drug Discovery Laboratory, Department of Pharmacology, Faculty of Pharmacy, Bezmialem Vakif University, Fatih, Istanbul 34093, Turkey; aakdemir@bezmialem.edu.tr; 5 Department of Life and Environmental Sciences, Unit of Pharmaceutical, Pharmacological and Nutraceutical Sciences, University of Cagliari, University Campus, S.P. n° 8, Km 0.700, I-09042 Monserrato, Cagliari, Italy; vonnis@unica.it; 6 Department of Pharmaceutical Chemistry, Kafrelsheikh University, Kafrelsheikh 33516, Egypt; wagdy2000@gmail.com; 7 Institute of Biosciences and Bioresources—National Research Council, via Pietro Castellino 111, 80131 Napoli, Italy; clemente.capasso@ibbr.cnr.it; 8 Institute of Biostructures and Bioimages—National Research Council, 80131 Napoli, Italy; gdesimon@unina.it (G.D.S.); marmonti@unina.it (S.M.M.); 9 Department of Pharmacy, “G. d’Annunzio” University of Chieti-Pescara, Via dei Vestini 31, 66100 Chieti, Italy; simone.carradori@unich.it; 10 School of Chemistry, University of New South Wales, 1466 Sydney, Australia; w.donald@unsw.edu.au; 11 Department of Integrative Oncology, BC Cancer Research Centre, Vancouver Vancouver, BC V5Z 1L3, Canada; sdedhar@bccrc.ca

**Keywords:** carbonic anhydrase, hypoxia, pH regulation, inhibitor, sulfonamide, SLC-0111

## Abstract

The tumor microenvironment is crucial for the growth of cancer cells, triggering particular biochemical and physiological changes, which frequently influence the outcome of anticancer therapies. The biochemical rationale behind many of these phenomena resides in the activation of transcription factors such as hypoxia-inducible factor 1 and 2 (HIF-1/2). In turn, the HIF pathway activates a number of genes including those involved in glucose metabolism, angiogenesis, and pH regulation. Several carbonic anhydrase (CA, EC 4.2.1.1) isoforms, such as CA IX and XII, actively participate in these processes and were validated as antitumor/antimetastatic drug targets. Here, we review the field of CA inhibitors (CAIs), which selectively inhibit the cancer-associated CA isoforms. Particular focus was on the identification of lead compounds and various inhibitor classes, and the measurement of CA inhibitory on-/off-target effects. In addition, the preclinical data that resulted in the identification of SLC-0111, a sulfonamide in Phase Ib/II clinical trials for the treatment of hypoxic, advanced solid tumors, are detailed.

## 1. Introduction

The microenvironment of tumor cells is different from that of normal cells and plays a crucial role in shaping the behavior of tumors, which, in turn, frequently influences treatment outcomes as well as treatment strategies [1,2]. Many decades ago, Warburg [3] recognized this phenomenon, which later became known as the “Warburg effect” and constitutes a hallmark of many cancers: these cells are hypoxic, more acidic than normal, and possess a dysregulated glucose (and not only glucose) metabolism [3,4]. In the last 20 years, it became obvious that the orchestrators of all these phenomena are transcription factors called hypoxia-inducible factor 1 and 2 (HIF-1/2). The understanding of the intricate biochemical and physiological processes by which HIF-1/2 sense tumor oxygen levels and regulate genes involved in metabolism (such as the glucose transporters), pH regulation (such as monocarboxylate transporters, MCTs; and carbonic anhydrases, CAs), and angiogenesis (vascular endothelial growth factor (VEGF)) led to the award of the 2019 medicine Nobel prize to three scientists who contributed significantly to the field, Kaelin [5], Ratcliffe [6], and Semenza [7,8]. Their crucial discoveries and those from many other laboratories [9,10,11,12,13,14,15,16,17] established the basis for exploiting the tumor microenvironment abnormalities for developing a new generation of antitumor therapies and drugs, which should specifically target tumor cells without relevant toxicity to normal cells and tissues [18,19].

As a result of HIF-1/2 activation, two of the proteins that were identified to be highly overexpressed in many tumor types were the CA isoforms, CA IX and XII [9,10,11,12,13,14,15,16,17,18,19]. CAs are a superfamily of metalloenzymes present in all kingdoms of life that catalyze the conversion of CO_2_ to bicarbonate and protons [20,21,22,23,24,25,26]. There are 15 CA isoforms that are known in humans (h); i.e., hCA I - hCA XIV, including two V-type isoforms, hCA VA and hCA VB) [27,28,29,30]. The field of CAs and their inhibitors were recently reviewed and will not be discussed in detail here [19,20,21,22,23,24]. Briefly, the X-ray crystal structures are available for the two tumor-associated isoforms hCA IX and XII [25,26], as well as for many other members of the human CA family [27,28,29,30]. Such structures of the enzymes alone and in complex with many types of inhibitors have been highly relevant for the design of compounds with a range of applications, not only in the anticancer field but also in the renal, central nervous system, ophthalmologic, obesity, and other medical fields [31,32,33,34,35,36,37,38,39]. Furthermore, recently, CA inhibitors were shown to be of potential use in the management of cerebral ischemia, neuropathic pain, and arthritis [40,41,42,43,44], which are conditions for which this class of pharmacologic agents was previously considered inappropriate. However, the attentive search for novel classes of compounds with efficacy and selectivity for the different isoforms involved in these quite diverse conditions resulted in proof of concept studies, which have suggested that all catalytically active hCA isoforms may be considered interesting drug targets.

## 2. Validation of CA IX/XII as Anticancer Drug Targets

### 2.1. Sulfonamides and Other Classes of CA inhibitors (CAIs): Selectivity for Tumor-Associated vs. Cytosolic Isoforms

hCA IX was discovered in 1994 by Pastorekova’s group [45] and hCA XII in 1998 by Türeci et al. [46]. Both isoforms were shown to be extracellular, multi-domain proteins, with the enzyme active site situated outside the cell [45,46]. Years later, with the demonstration that both enzymes are activated through the HIF-1/2 pathway, a reason for this localization became obvious: both enzymes, together with a range of other proteins that will be not discussed here, are involved in the pH regulation and metabolism of the cancer cell [1,2,47,48,49,50,51,52,53]. Both enzymes possess significant catalytic activity for the CO_2_ hydration reaction [19,20,21].

**Figure 1:**
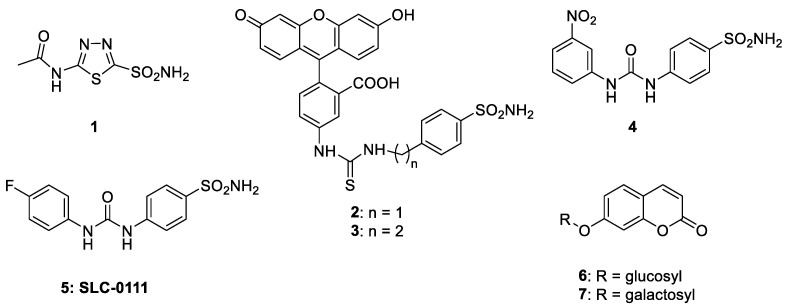
Carbonic anhydrase inhibitors (CAIs) that were crucial for validation of CA IX/XII as antitumor targets: acetazolamide **1** is the classical inhibitor in clinical use for decades; the fluorescein-derivatized sulfonamides **2** and **3** were used in the proof of concept studies to demonstrate the involvement of CA IX in acidification of the external pH of the tumor cell [47,48,49,50], the ureido-substituted-sulfonamides **4** and **5** were among the first CAIs to show significant antitumor effects in animal models of hypoxic tumors [51,52], together with coumarin inhibitors **6** and **7** [53].

The hypothesis that interfering with the activity of such proteins may have anticancer effects was proposed by Pouysségur in 2006 [18], but no selective inhibitors for cancer-associated CAs or other proteins involved in the regulation of the tumor microenvironment (MCTs, bicarbonate transporters, or Na+/H+-exchangers, etc.) were available at that time. Thus, a program for developing CA IX/XII selective compounds was initiated in some of our (CT Supuran and S Dedhar) laboratories. Acetazolamide **1** (Figure 1), the classical sulfonamide inhibitor, was the starting point [54,55]. Indeed, primary sulfonamides [56,57,58] such as acetazolamide were known for decades to potently inhibit CAs, as they bind as anions (RSO_2_NH^−^) to the metal ion in the enzyme active site, as shown in a classical crystallographic study from Liljas’ group [59]. However, acetazolamide is a non-selective CAI [20], which potently inhibits most human CA isoforms and is, thus, not an appropriate compound for designing isoform-selective compounds. The tail approach was thus employed for developing CA IX/XII-selective inhibitors [60]. The idea is very simple: attach moieties that may induce the desired properties (e.g., enhanced hydrosolubility) to scaffolds of simple sulfonamides (e.g., sulfanilamide, metanilamide, and their derivatives; 5-amino-1,3,4-thiadiazole-2-sulfonamide, etc.) that may lead to interactions with the external part of the CA isoforms active site, which is the region at the entrance of the cavity that is more diverse than the active site residues between the 12 catalytically active human isoforms [60,61]. Detailed kinetic and crystallographic studies were performed on a large number of derivatives (more than 10,000 sulfonamides were synthesized and investigated in our laboratories). This research demonstrated that it was possible to design CAIs selective for the various isoforms by tailoring the dimensions (length, bulkiness, etc.) as well as the chemical nature of the various tails [60,61,62,63,64,65,66,67,68,69]. For example, the fluorescein-derivatized sulfonamides **2** and **3** were only mildly selective for CA IX vs. CA II [47,48], but they were highly useful for understanding the role of CA IX in tumor pH regulation [47,48,49,50]. In contrast, the ureido-substituted sulfonamides **4** and **5** were members of a congeneric series in which many CA IX/XII selective inhibitors were discovered [51,52], and their selectivity for the tumor-associated vs. the cytosolic isoforms was explained by detailed X-ray crystallographic data [70,71]. Another highly interesting approach was reported by Neri’s group [72,73,74], who designed DNA-encoded libraries of sulfonamides and identified highly potent in vitro CA IX inhibitors in a series of very interesting papers [75,76,77].

Thus, by 2009–2010, sulfonamide CAIs targeting CA IX with high specificity were available [78], which fostered renewed interest in targeting not only primary tumors but also metastases [79,80,81]. However, in parallel with these studies mentioned above, the last decade also brought important developments in the discovery of non-sulfonamide CAIs. The first highly relevant new class of CAIs is represented by the coumarins [82,83,84,85]. The coumarins were shown to act as “prodrug inhibitors”: they undergo CA-mediated hydrolysis of the lactone ring (as in the compounds **6** and **7** of Figure 1), which generates the de facto inhibitor, belonging to the 2-hydroxycinnamic acid class of compounds. Extensive kinetic, crystallographic, and synthetic efforts led to a thorough understanding of this innovative inhibition mechanism [82,83,84,85]. In fact, the 2-hydroxycinnamic acids formed by coumarin hydrolysis do not interact with the catalytic metal ion but instead bind at the entrance of the active site cavity, in a highly variable region of the different CA isoforms, which explains the very isoform-selective inhibitory effects of many representatives of this class of CAIs [82,83,84,85]. Furthermore, this new CA inhibitory class inspired the discovery of many other chemotypes, such as the sulfocoumarins and the homosulfocoumarins by Zalubovskis group [86,87,88], the homocoumarins [89], the thiocoumarins by Ferraroni et al. [90], etc.

By comparing the sulfur-based, carbon-based [91], and phosphorus-based zinc-binding groups, a range of novel classes of inhibitors that interact with the zinc ion were reported, including phosphonamidates [92], dithiocarbamates [93], monothiocarbamates [94], xanthates and trithiocarbonates [95], selenols [96,97], carboxylates [98] and hydroxamates [99], benzoxaboroles [100,101,102], as well as carbamates [103]. Additional inhibitors that have been developed exhibit a different inhibition mechanism: they anchor to the zinc-coordinated water molecule [104,105]. Polyamines, such as spermine and its derivatives [104], and the simple phenol [105] and many of its derivatives [106,107,108,109] together with polyphenols [110] inhibit CAs in this way. This inhibition mechanism is also shared by the sulfocoumarins, as demonstrated by Zalubovskis group [86] by using kinetic and crystallographic studies. However, probably, one of the strangest CA inhibition mechanism is the one reported by De Simone’s and Carradori’s group for a benzoic acid derivative that binds outside the active site cavity [111].

### 2.2. Measurement of Inhibition Efficacy

All these inhibitors discussed above and many others that are not mentioned here have been profiled for their inhibition of many CA isoforms by using a stopped-flow CO_2_ hydrase assay, which was originally reported in 1971 by Khalifah [112] and validated by others as a rapid method for determining the enzyme kinetics and inhibitory constants of various classes of compounds against different CAs [113,114,115,116,117,118]. In a recent paper by Jonsson and Liljas [119], some inhibition data from several papers from the Supuran group were considered. Jonsson and Liljas queried the enzyme and substrate concentrations that were used in some experiments. Many of the early stopped-flow assay papers cited above [113,114,115,116,117,118], which are not from Supuran’s group but from several well-established laboratories, provided exactly the same level of information. That is, the range of CO_2_ concentrations at which the experiments were performed was reported, without detailing enzyme concentrations. On the contrary, in many of our papers, the enzyme concentrations are reported either for the CO_2_ hydrase or esterase assays [60,62,68,69,120,121,122,123,124,125,126,127], and they range between 3.5 and 14 nM. The vast majority of the analyses performed by Jonsson and Liljas [119] are based on supplementary information that was reported in six of our papers (two of the other papers that they considered in the analysis contained CA inhibitory data obtained from a completely different laboratory). The presumed lack of precision results from erroneously typed concentrations for the enzymes in one of the analyzed paper’s supplementary information [128]. That is, a concentration of 10^−7^ M was erroneously written, which is, in fact, the stock solution of enzyme and not the enzyme concentration at which the measurements were performed. As mentioned above, in most of our experiments, we work at enzyme concentrations ranging from 3.5 and 14 nM, and sometimes even lower when exceedingly strong inhibitors are analyzed. Jonsson and Liljas [119] also raised a query about the enzyme inhibition curves from the supplementary information of another paper [129]. For these figures, the uncatalyzed reaction was not subtracted in the curves plotted in the supplementary figures, but those values were automatically subtracted when the IC_50_ was calculated due to the use of the algorithm in the computer software that is used for the stopped-flow instrument. Despite these minor errors (now corrected through submission of errata), the inhibitory constants (*K*_i_s) determined by the University of Florence stopped-flow kinetic measurements of CA enzyme inhibition have been validated by native mass spectrometry (MS) measurements performed in another laboratory on a number of sulfonamides, which gave results in excellent agreement with the kinetic inhibition data [130,131]. Dissociation constants (*K*_d_s) were obtained from native mass spectrometry measurements of buffered aqueous solutions containing hCA I and hCA II with acetazolamide, ethoxzolamide, brinzolamide, furosemide, dichlorophenamide, and indapamide, which are well known CAIs [20]. *K*_d_ values were obtained by either measuring individual ligand–protein interactions in single experiments or simultaneously in competition experiments (Table 1) [130]. *K*_d_ values are equivalent to *K*_i_ values for inhibitors that bind at the site of the substrate, which is the case for sulfonamide CAIs. The agreement between the native MS measurements and those for the stopped-flow kinetic assay was excellent. For example, the measured *K*_d_ values of all nanomolar inhibitors were within 30% of the *K*_i_ values obtained by stopped-flow kinetic experiments. Moreover, *K*_d_ values obtained by native MS for the micromolar inhibitors were within 50% of the *K*_i_ values. More recently, native MS was used to measure the *K*_d_ values of 15 perfluoroalkyl substances with either carboxylate, sulfate, or sulfonamide zinc-binding groups to hCA I and hCA II [131]. The 30 measured *K*_d_ values obtained from native MS measurements were also in excellent agreement with the corresponding *K*_i_ measurements (data not shown here) [131]. In fact, the *K*_d_ values were within 37% of the *K*_i_ values. The native MS approach to measuring *K*_d_ values has also been validated for ligand–DNA interactions compared to other well-established biophysical methods (isothermal titration calorimetry and differential scanning calorimetry) [132].

Overall, these native MS data strongly support that *K*_i_ values can be obtained using the stopped-flow kinetic method for a range of different types of CAIs with sufficient accuracy for drug discovery and development applications.

### 2.3. Drug Design Studies Using SLC-0111 as Lead Compound

Compound **5**, SLC-0111, possesses a variable affinity for the diverse hCA isoforms and selective inhibitory action toward tumor expressed CA isoforms IX and XII over the off-target ubiquitous hCA I and hCA II [51,52,70,81]. Compound **5** is characterized by the presence of the ureido functionality as a linker between the benzenesulfonamide fragment and the tail of the inhibitor. By means of X-ray crystallography on SLC-0111, it was demonstrated that the reason for the isoform selectivity is the ureido linker, which allows great flexibility to the CAI “tails” that may adopt a range of conformations and participate in different interactions within the enzyme active site [52,70]. Such different favorable/unfavorable contacts between the inhibitor tail and the enzyme active site lead to different inhibition profiles for the entire class of sulfonamides to which SLC-0111 belongs [52,70]. The different tail orientations allow the specific interactions between the inhibitor tail and amino acid residues at the entrance of the active site cavity, which is the most variable region in the various α-CA isoforms with medicinal chemistry applications, such as, for example, CA I, II, IX, and XII [19,20,21,138,139,140,141,142]. SLC-0111 binds selectively to hCA IX coordinating through the SO_2_NH^−^ moiety (the deprotonated form of sulfonamide group) to the positively charged Zn(II) ion in the CA active site. In addition, a second strong contact with the Zn(II) ion involves oxygen of the sulfonamide. In contrast, these interactions are either weak or absent in the case of the hCA II isoform. The large difference in the electrostatic interactions accounts for the selectivity of the ligand to hCA IX. It was suggested that the potency of SLC-0111 against isoform IX is due to the hydrophobic contacts, whereas the selectivity is due to the electrostatic interactions, by means of computational approaches [143].

As a front-runner selective inhibitor for the tumor-associated isoform CA IX, and being currently in Phase Ib/II clinical trials, SLC-0111 has been utilized as a lead CAI for the development of novel promising small molecules with selective inhibitory activity toward CA IX, and with good druggability and lead-likeness characters. Several drug design approaches have been utilized to develop a range of new SLC-0111 analogs. The following subsection will present an overview of most of these drug design approaches.

#### 2.3.1. Modification of the SLC-0111 Benzenesulfonamide Moiety

**Figure 2:**
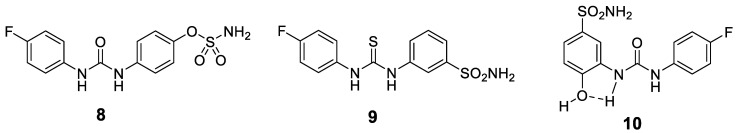
Chemical structures for SLC-0111 analogs (**8**–**10**) developed through modification of the SLC-0111 benzenesulfonamide moiety.

In 2012, Gieling et al. developed new sulfamate-based SLC-0111 analogs via replacement of the sulfamoyl zinc-binding group (ZBG) in SLC-0111 with the sulfamate functionality (Compound **8**, Figure 2). The reported sulfamate derivatives in this study displayed selective CA IX/XII inhibition, as well as inhibited migration and spreading of breast cancer MDA-MB-231 cells. One of these sulfamate-based CAIs efficiently inhibited the development of MDA-MB-231 metastases in the lung without signs of toxicity [144].

In another study, Carta et al. reported new SLC-0111 regioisomers (Compound **9**, Figure 2). The obtained results revealed that shifting of the sulfamoyl ZBG in SLC-0111 from the para- to meta-position elicited a decrease in the effectiveness toward hCA IX with significant improvement of the inhibitory action against hCA II, which was detrimental to the selectivity profile for this regioisomer [145].

On the other hand, Bozdag et al. reported the design and synthesis of new SLC-0111 congeners incorporating a 2-aminophenol-4-sulfonamide moiety to control the tail flexibility, (Compound **10**, Figure 2). The phenolic OH was able to establish an intra-molecular five-membered ring with the ureido NH group, which may provoke a C2-N’rotational restriction leading to a 20-fold enhanced hCA II/hCA IX selectivity ratio in comparison to SLC-0111 [146].

#### 2.3.2. Modification of the SLC-0111 Ureido Linker

**Figure 3:**
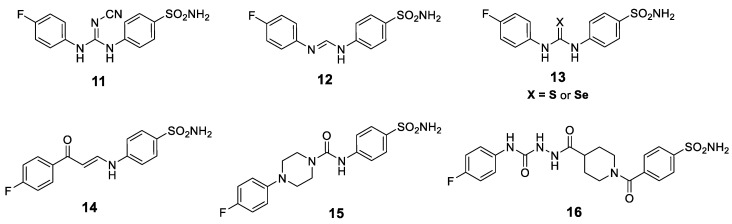
Chemical structures for SLC-0111 analogs (**11**–**16**) developed through modification of the SLC-0111 ureido linker.

Akocak and co-workers adopted a bioisosteric replacement approach in order to replace the SLC-0111 ureido linker with a cyanoguanidine (Compound **11**, Figure 3) or 1,3-triazene (Compound **12**, Figure 3) linker. The developed *N*,*N*′-diaryl cyanoguanidines and 1,3-diaryltriazenes in both studies emerged as selective hCA II inhibitors [147,148,149].

The discovery of other SLC-0111 analogs was continued through two new studies that described the synthesis of novel sets of thioureido and selenoureido CAIs. In these studies, the SLC-0111 ureido oxygen was replaced with a sulfur or selenium atom (Compound **13**, Figure 3) [150,151]. The inhibition profile for both thioureido and selenoureido SLC-0111 congeners showed a loss of selectivity for the inhibition of the cancer-associated cytosolic *h*CA isoforms [150,151]. Moreover, Eldehna et al., in 2019, developed a series of 3/4-(3-aryl-3-oxopropenyl)aminobenzenesulfonamide derivatives as novel SLC-0111 enaminone analogs (Compound **14**, Figure 3). All the reported enaminones exhibited good selectivity toward hCA IX over hCA I and II. The structure-activity relationship (SAR) outcomes highlighted the significance of the incorporation of a bulkier aryl tail such as the 2-naphthyl ring [151].

In order to manipulate the flexibility of the SLC-0111 ureido linker, the urea linker outer nitrogen atom was incorporated into a piperazine ring to produce rigidified SLC-0111 analogs (Compound **15**, Figure 3). The rigid congeners displayed a reduction of CA IX/CA II selectivity, although some nanomolar CA IX inhibitors were obtained [152].

Furthermore, to better understand the importance of a rigid heterocyclic scaffold, the piperazine ring was substituted with piperidine (Compound **16**, Figure 3). In this compound series, a hydrazinocarbonyl-ureido moiety was introduced for the tail of the inhibitors. The NH group of the hydrazide moiety may provide a supplementary H-bond donator, which can better interact with the aminoacidic residues in the hydrophilic region of the active site. Depending on the substitution pattern at the piperidino ring, several hydrazidoureidobenzensulfonamides inhibited CA IX at low nanomolar concentrations [153].

#### 2.3.3. Modification of the SLC-0111 Tail

**Figure 4:**
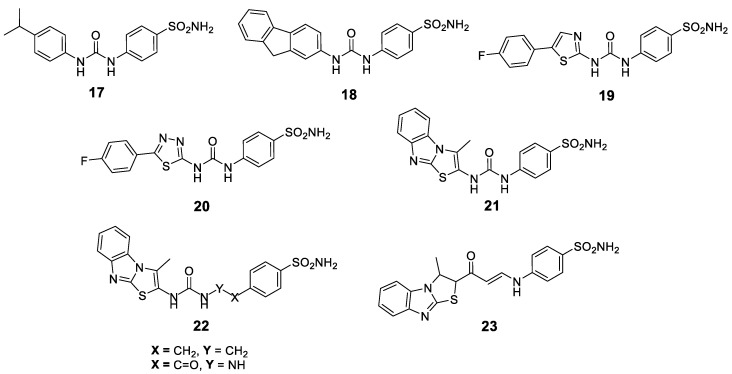
Chemical structures for SLC-0111 analogs (**17**–**23**) developed through modification of the SLC-0111 tail.

**Figure 5:**
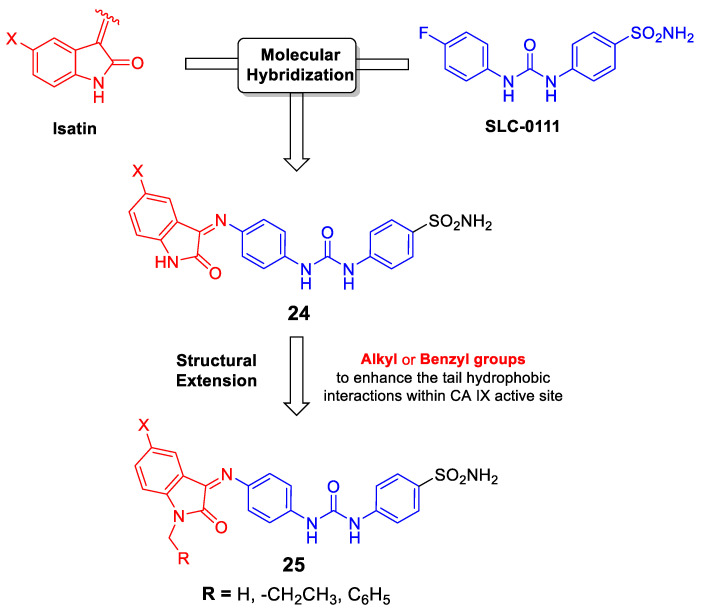
Design of the *N*-(un)substituted isatins-SLC-0111 hybrids **24** and **25** [157,158].

The replacement of the 4-fluorophenyl tail with a 3-nitrophenyl group afforded low nanomolar CA IX/XII inhibitor with good selectivity for the transmembrane over the cytosolic isoforms (Compound **4**, Figure 1). The same SLC-0111 analog significantly inhibited the formation of metastases by the highly aggressive 4T1 mammary tumor cells [52]. Furthermore, novel SLC-0111 congeners were synthesized either via grafting different substituents within the phenyl tail, rather than *p*-fluoro (Compound **17**, Figure 4) or via replacement of the 4-fluorophenyl moiety with polycyclic tails (Compound **18**, Figure 4) [52]. On the other hand, replacement of the 4-fluorophenyl tail of SLC-0111 with 4-arylthiazole (Compound **19**, Figure 4) and 5-arylthiadiazole (Compound **20**, Figure 4) successfully improved the inhibitory activity toward hCA IX. Unfortunately, the most active thiadiazole analogs showed a decrease of the hCA IX/II selectivity as compared to SLC-0111 [154]. Another recent study has utilized the bioisosteric replacement approach to design and synthesize new SLC-0111 analogs featuring 3-methylthiazolo[3,2-*a*]benzimidazole moiety as a tail that connected to the zinc-anchoring benzenesulfonamide moiety via a ureido linker (Compound **21**, Figure 4). Thereafter, the ureido linker was either elongated (Compound **22**, Figure 4) or replaced by an enaminone linker (Compound **23**, Figure 4) [155]. The results obtained from the stopped-flow CO_2_ hydrase assay elucidated that three compounds possessed single-digit nanomolar CA IX inhibitory action with good selectivity profile toward hCA IX over hCA I and II. Moreover, these compounds exerted effective anti-proliferative and pro-apoptotic activities toward breast cancer MCF-7 and MDA-MB-231 cell lines. It is worth noting that the molecular docking analysis disclosed that thiazolo[3,2-*a*]benzimidazole moiety is a good bioisoster for the SLC-0111 phenyl tail due to its ability to establish many hydrophobic interactions within the hCA IX and XII active sites, as well as involving the sp^2^ nitrogen of the tricyclic ring in hydrogen bonding. The fluorine atom from SLC-0111 was also replaced by metal-complexing polycyclic amines, which coordinate positron-emitting (PET) metal ions (e.g., ^111^In and ^90^Y) for PET imaging [156].

#### 2.3.4. Development of SLC-0111 Hybrids

In 2016, Eldehna et al. has utilized a hybrid pharmacophore approach to merge the pharmacophoric elements of the isatin, a privileged scaffold in cancer drug discovery, and SLC-0111 in a single chemical framework to develop a new series of novel isatins-SLC-0111 hybrids (Compound **24**, Figure 5) [157]. Whilst most of the prepared hybrids exerted excellent inhibition for hCA XII in the sub- to low-nanomolar range, they weakly inhibited hCA IX. Thereafter, a structural extension approach has been exploited through *N*-alkylation and *N*-benzylation of the isatin moiety in order to enhance the hydrophobic interactions of the tail within the CA IX binding site, with the prime goal of improving the activity and selectivity toward the CA IX isoform (Compound **25**, Figure 5). As planned, an improvement of hCA IX inhibitory activity for the hybrids, in comparison to *N*-unsubstituted counterparts (Compound **24**), was achieved. Furthermore, one of the developed *N*-substituted isatin-SLC-0111 hybrids showed potent VEGFR-2 inhibitory activity and good anti-proliferative action toward breast cancer MDA-MB-231 and MCF-7 cell lines under hypoxic conditions. Furthermore, it disrupted the MDA-MB-231 cell cycle via alteration of the Sub-G_1_ phase and arrest of G_2_-M stage, as well as, it resulted in a significant increase in the percent of Annexin V positive apoptotic cells [158].

## 3. In Vivo Studies, Preclinical and Clinical Trials of CA IX/XII Inhibitors

Overall, the methodology used to determine the inhibitory constants for the various compounds determined in the papers discussed above, and validated by independent technologies (see above), represent bona fide, accurate data, which in no way compromise the development of CA IX and CA XII-specific inhibitors for validation in preclinical cancer models and in clinical trials, based on the vast biological literature demonstrating CA IX/CA XII as promising cancer therapeutic targets in hypoxic solid tumors.

The compounds from which SLC-0111, the lead clinical CA IX/CA XII inhibitor, was derived (**5** in Figure 1), were identified by the Supuran group [51,52]. These ureidobenzene sulfonamide compounds were then assessed for “druggability” criteria, including ADME (Absorption, Distribution, Metabolism, Excretion) analysis, from which SLC-0111 was selected for further in vitro and in vivo analysis in appropriate cancer models. The evaluation of CA IX/CA XII inhibitors as anticancer compounds takes a different path from the development of other cytotoxic anticancer drugs. This is because the targets, CA IX/CA XII are only expressed within the hypoxic niches of solid tumors and may represent a minor portion of the total tumor cell population. However, these hypoxic cells have the properties for self-renewal [159,160,161], migration/invasion [162,163,164], and survival in an acidic tumor microenvironment [163,164,165,166,167,168] and significantly contribute to resistance to chemo-, radiation, and immunotherapies. Thus, CA IX/CA XII inhibitors are not likely to have a major effect on tumor growth and metastasis by themselves as mono-therapeutics but need to be used in combination with chemo-, radiation-, and immunotherapies to eliminate resistant populations and for maximum durable suppression of tumor growth and metastasis.

Indeed, the extensive preclinical models carried out by several independent groups with the lead CA IX/XII inhibitors, including SLC-0111, have demonstrated that the use of such inhibitors in combination with chemotherapy agents [51,169,170,171], immunotherapy [172,173,174], and radiotherapy [173,174,175,176] is highly important and desirable for sustained therapeutic response. The extensive studies reported in these and other papers, utilizing multiple in-depth in vivo models, provided solid positive preclinical data to warrant the initiation of Phase 1 clinical trials in 2014, of which a Phase 1 safety trial with SLC-0111 (as a monotherapeutic agent), has been completed [177] and a Phase 1b trial is currently underway to evaluate SLC-0111 in combination with gemcitabine in metastatic pancreatic cancer patients whose tumors are CA IX positive (ClinicalTrials.gov Identifier: NCT03450018).

A multitude of other combination therapy studies has been performed with CA IX inhibitors, including SLC-0111, in combination with proton pump inhibitors [178], antimetabolites [179], cisplatin [180], APE1-Ref-1 inhibitors [181], and histone deacetylase (HDAC) inhibitors [182]. All these studies showed a synergistic effect between the CAI and the second antitumor agent, as well as the lack of endothelial toxicity [183].

Other groups also used SLC-0111 in various biomedical studies in which selective inhibition of some CA isoforms was needed. These include the effects on prostate cancer cells of SLC-0111 alone or in combination with daunorubicin [184], radiobiological effects of CA IX inhibition in human breast cancer cells [185], microvascular endothelial cell pH regulation [186], glycolysis and migration suppression in pulmonary microvascular endothelial cells [187], and the involvement of CA isoforms in mitochondrial biogenesis and in the control of lactate production in human Sertoli cells [188]. All these studies confirm the usefulness of this clinical candidate in tumors and other biomedical conditions.

## 4. Conclusions

The tumor microenvironment is critical in cancer cell growth and can substantially influence the outcome of anticancer interventions. Transcription factors, such as HIF-1/2, can activate a number of key genes including those involved in tumor pH regulation. The human carbonic anhydrase isoforms CA IX and XII have active roles in regulating the extracellular pH in cancers including in advanced solid metastatic tumors. As a result of careful preclinical studies involving X-ray crystallography [189] and the screening of tens of thousands of potential inhibitors [190,191,192,193] by use of a functional kinetic enzyme inhibition assay based on stopped-flow kinetic measurements, key lead compounds for CA IX and XII, including the sulfonamide CA inhibitor SLC-0111, have been discovered, developed, and validated. The results from the stopped-flow kinetic enzyme inhibition assay are in excellent agreement with: (i) orthogonal native mass spectrometry measurements that can be used to directly measure protein–ligand interactions and (ii) kinetic inhibition measurements that do not involve a stopped-flow apparatus. Thus, the stopped-flow kinetic method has been used to screen tens of thousands of potential CA inhibitors with sufficiently high accuracy for drug discovery and development applications. Based on the vast literature demonstrating the development and validation of specific CA IX and CA XII inhibitors, including in preclinical cancer models and in clinical trials, these two enzymes are promising cancer therapeutic targets in advanced, hypoxic solid tumors. We anticipate that the use of selective CA IX/XII inhibitors, such as SLC-0111, will be beneficial in combination with chemo-, radiation-, and immunotherapies to eliminate resistant cancer cell populations and for maximum durable suppression of tumor growth and metastasis.

## Figures and Tables

**Table 1:**
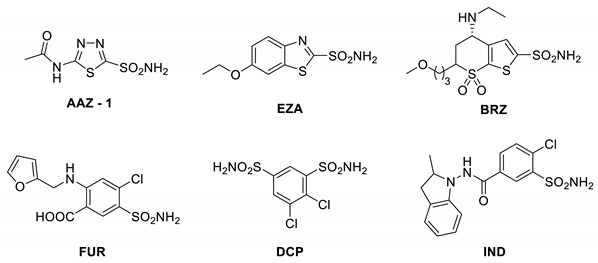
Measured dissociation constants (μM) of six sulfonamides to human (h)CA I and hCA II using native mass spectrometry with nanoscale ion emitter tips in individual ligand–protein binding experiments (Single) and simultaneously in a competition experiment (Competition). Data adapted from Reference [130].

	hCA I	hCA II				
Inhibitor	Single	Competition	Literature	Single	Competition	Literature
Acetazolamide (AAZ)	0.24 ± 0.02	0.29 ± 0.03	0.25 ^a^ [133]	0.015 ± 0.001	0.022 ± 0.002	0.012 ^a^ [133]
Ethoxzolamide (EZA)	0.014 ± 0.002	0.016 ± 0.004	0.025 ^a^ [133], 0.009 ^b^ [134]	0.010 ± 0.001	0.014 ± 0.002	0.008 ^a^ [133]
Brinzolamide (BRZ)	1.06 ± 0.05	1.12 ± 0.06	0.73 ± 0.04^a^ [135]	0.005 ± 0.001	0.007 ± 0.001	0.003 ^a^ [135]
Furosemide (FUR)	0.055 ± 0.005	0.048 ± 0.006	0.062 ^a^ [130]	0.098 ± 0.009	0.110 ± 0.010	0.065 ^a^ [135]
Dichlorophenamide (DCP)	1.3 ± 0.1	1.4 ± 0.1	1.2 ^a^ [136]	0.027 ± 0.002	0.035 ± 0.002	0.038 ^a^ [136]
Indapamide (IND)	9.2 ± 0.3	9.5 ± 1.0	51.9 ^a^ [137]	3.22 ± 0.20	3.32 ± 0.31	2.52 ^a^ [137]

^a^ U. Florence stopped-flow kinetic inhibition constant measurements (μM) [68,69,82,120,121,122,123,124,125,126,127]. ^b^ U. Florida buffer indicator kinetic method measurements [134].
